# Our Hospitals' Christmas, 1918

**Published:** 1919-01-04

**Authors:** 


### OUR HOSPITALS' CHRISTMAS, 1918.

### Its Inspiring Influence and Joyous Atmosphere.

GUY'S HOSPITAL was one of the earliest, if not actually the first great Metropolitan hospital to make Christmas Day within its walls so bright and wholeheartedly joyous, with a genial, merry, hearty outpouring of personal service everywhere, that those who found themselves present in the institution breathed a new atmosphere which aroused their best instincts and gave them a new outlook on life and their own responsibilities. Every healthy person was thus afforded an opportunity to realise the privilege they had to give of themselves to lighten the burdens of sick people, and so render thanks for the blessings of their own health. The system on which Christmas is commemorated and spent at Guy's Hospital has a tendency to raise the standards of its workers and to bring to the service of the sick each year more and better means, involving the development of artistic qualities which have given an added charm to the decorations, and have made the wards at Christmas-time places where every visitor could find much to instruct and inspire, where everybody engaged in the service puts forth every ounce of the best of which they are capable, so that each patient grows cheerful and is taken out of himself to a higher atmosphere, with results which are wholly beneficial and often inspiring too. This year we have to welcome a brief outline of some of the more striking features of the ward decorations, which we have great pleasure in publishing as we received it. Some of the wards in the artistic sense were beautiful and exhibited considerable talent on the part of the sisters and those who helped in their decoration. The Astley Cooper ward, to take one example, was decorated throughout with poinsettias. The electric-light shades were made to take this form, and the whole ward was surprisingly beautiful, especially when the electric light was turned on. The effect was heightened from the circumstance that the main accident ward on the ground floor was so excellently kept that its floors and passages attracted the eye and excited commendation and wonder. Here was proof indeed that a hospital affords every worker within its walls, from the humblest to the highest, unique opportunities for living up to the highest standards of which they are capable. To realise the beauties each reader must recall the fact that the poinsettia is a plant much cultivated in conservatories. It is conspicuous for the large scarlet floral leaves surrounding its crowded yellowish cymes of small flowers, and is much used for decoration. The first Christmas after the war produced a famine in flowers and decorations everywhere. Yet a horticulturist on entering the Astley Cooper ward, where the specimens of poinsettia were so prodigally displayed throughout the ward, exclaimed: "Good gracious, they must have spent a small fortune." On closer examination, though it was often difficult to convince oneself of the fact, not a single poinsettia flower was used in the decoration. The sister thought out her scheme of decoration, and with the aid of friends found the materials, and supplied or obtained the artistic help which enabled lifelike poinsettias to be produced in considerable numbers, from paper and other materials. If the Horticultural Society in the winter were to give an exhibition of flowers, and to throw it open to the sisters of Guy's Hospital, their combined artistic ability and good taste might produce an exhibition which would attract the town, provided it was announced as a Christmas exhibition of available flowers, and the means and materials which formed them were withheld from the public announcement. Wonderful is the only word which correctly defines the results achieved, not only in the Astley Cooper ward, but in several others which we wish we had space to describe in detail. Charity ward was strikingly decorated with cherries and pink hand-painted silk lampshades, which proved very effective and attractive. Every cherry had to be made by hand, and some 3,000 were required for the purposes of the decorations.

The Sir Alfred Fripp wards are Charity, Cornelius, and Luke, and every Christmas, commencing on Christmas Eve, and extending through the whole of Christmas afternoon, Sir Alfred devotes himself with no unlavish hand to the joyous task of helping the sister and many willing helpers to make every patient, from the child to the adult, spend a Christmas, the memory of which will linger with them for many years. On Christmas Eve he arranged a concert in which several well-known actors and actresses, whose names are given elsewhere, gave invaluable personal service.

It has been our privilege to spend many Chrismases in the wards of Guy's Hospital. This year, in the days preceding Christmas, we were overweighted with continuous work, to which nature succumbed, but the memories of the past stimulated an ever-growing desire to get to Guy's Hospital, and on Christmas Day, once again, we found ourselves in the wards. We came away thankful to have enjoyed the privilege, and grateful indeed to the noble army of students, members of the staff and employees, with their friends, who made Guy's Hospital a place of inspiration and helpful uplifting for everyone possessed with apprehension and the power to appreciate the atmosphere created by the highest standard of human efforts its men and women were capable of. We have one wish still ungratified, and it is this: that the Christmas spirit which has been developed in so many hospitals throughout the land could be quickened everywhere so that Christmas Day and Christmas week should be made days of inspiration and wholehearted enjoyment of the spirit of Christmas at its best. We have many good friends at Guy's

**OUR HOSPITALS' CHRISTMAS, 1918**—(*continued*).

Hospital, and one regret is that from its size it is impossible to find and converse with everyone of them every time we have the privilege of finding ourselves within its walls.

We append the brief account, for which we are indebted to one of the sisters, whom gratitude would name, but whose modesty has deprived us of this privilege.

### Guy's Wards Seen From Within.

For the last four years Christmas has not been without its background of care and anxiety with the overhanging shadow of the great war. This year, however, one and all at Guy's Hospital were able to throw off the burden of restraint, and consequently something of the old time spirit permeated the many and varied festivities throughout the week. On Christmas Eve the concert kindly arranged by Sir Alfred Fripp for the benefit of the patients in Charity, Cornelius and Luke wards was given and very much appreciated, several well-known actors and actresses, including Miss Winifred Barnes, Mr. Joseph Coyne, and others, coming and working strenuously prior to a heavy evening's work at the theatre. The decorations in Charity Ward of cherries and pink hand-painted silk lamp shades were very effective indeed. The wards, generally, were beautifully and elaborately decorated, and many original ideas were present. In the medical block, especially, Esther with its miniature gardens here and there, Queen and Victoria with its autumn scene. There was a refreshing fruit scene in Bright, looking so real as to be very tempting in the present day of prohibitive prices of fruit. Several other fresh ideas were seen in the shape of a Dutch landscape in Evelyn, with a Dutch garden ingeniously arranged; in Naaman, where the decorative scheme was a country scene represented by ponds filled with water-lilies and bulrushes, moss, and butterflies, suggesting a refreshingly peaceful atmosphere, and in Martha, where the decorations made their special appeal to the children, depicting the nursery rhyme—Oranges and Lemons—carried out very completely and prettily. There were so many novelties it was extremely difficult to single out any ward from another.

On Christmas Day the residents, in spite of their very heavy rush of work, were able to give a most excellent entertainment to the patients in every ward, devoting the whole of Christmas Day, as is their custom, to amuse the patients. They received more than their usual applause. Concerts and Christmas tea parties have been numerous throughout the week. On Christmas Day the tea given annually to the 700 out-patient children took place—the "nigger" troupe coming and delighting the children with their popular choruses and funny costumes. Every child received a toy from Father Christmas off two gigantic Christmas trees. In Lydia the in-patient children also had their Christmas tree, from which Father Christmas presented each child with toys. The last function, and one of the most important, is the evening concert held in Astley Cooper, followed by demonstrations in front of the hospital heralding in the New Year.

### St. Bartholomew's Hospital.

On Christmas morning Santa Claus and his assistant arrived at the hospital, visited the wards, and distributed presents to the patients. To be in keeping with his surroundings he had changed the fur on his robe into cotton-wool, replaced his sleigh by a wheeled stretcher, and a nurse (with unmistakably masculine feet and figure) led his donkey. Fred, the donkey, who had taken the place of the more conventional reindeer, was a bit unruly at times, the hind legs showing a tendency to dissociate from the front legs in accordance with the best pantomime traditions, but on the whole he was very docile and got on well with the children, although there was rather a panic among the younger ones when he first appeared. Later Fred desquamated and resolved himself into his constituent parts—two hot and dishevelled members of the resident staff.

In the afternoon there were entertainments in the wards. The Beery Bolsheviks, a troupe of endogenous origin, were a great success. Clad in red-stained operation gowns and red wigs and armed to the teeth, they conjured, sang clever and topical limericks, and acted a thrilling burlesque opera. As an example of their genius it may be mentioned that they found rhymes to such words as syringomyelia and hæmatoporphyrinuria! Such was their endurance that they gave eleven performances during the afternoon, every one of which was thoroughly enjoyed by the audiences. There were three other parties composed of ladies and gentlemen from outside, who very kindly gave up their afternoon to the hospital, and so with these and the usual extempore sing-songs the patients had plenty of amusement.

The wards were decorated, some with holly and evergreens, some—mostly in the military wing—with flags, others with tissue paper and fairy lamps, the colour schemes being all different and all pretty. It would be difficult indeed to say which was the best. In one ward was a large model of a battleship, with guns made of grey-painted test tubes, searchlights of pill-boxes, and cotton-reels as bollards. In another was a large Zeppelin suspended from the ceiling. Many had Christmas trees and all had tea going—a very different tea from the wartime ones of the last four years! so altogether the Peace Christmas was a great success.

### Queen Alexandra and Her Daughters at the London Hospital.

"N. B. M." writes: It was a "Victory Christmas" this year. At least, everyone felt that the war was over and it was a time that we could really rejoice. There were no thoughts of air raids, no anxious news expected from the "Front," the Food Controller had relaxed some of the stringent restrictions on Christmas fare, and, though there were still many of our friends absent, who in former years helped to make "the London" Christmas, we determined to use the talent we had to make it a real jolly time for all, and our efforts were not without success. The festivities really began at 11 o'clock on Christmas Eve, when the forty old night scrubbers were provided in the Out-Patient Department with a sumptuous tea of sausage rolls, cakes, buns, apples, and many other good things; they also had a warm shawl and two dried haddocks each, which they were allowed to take home with them. One old lady, recently pensioned after twenty years' faithful service, was the guest of the evening. She was asked how she intended getting home, as it was after midnight, and she came from a distance. She had no hesitation in replying "I shall be having a turn at polishing the House Governor's desk to-night," her old job, and how she polished it! Another woman was heard to say that she had only one complaint to make if she was asked, and "she would tell 'em that she had not been told she would get such a time or she would have brought bigger apron pockets"! The patients' Christmas started in the early morning. A procession of about

**OUR HOSPITALS' CHRISTMAS, 1918—(*continued*).**

140 sisters and nurses went through the wards singing carols to the accompaniment of violins. It was a pretty and impressive sight to see these sisters and nurses carrying coloured lanterns and singing as they passed through the wards. Shortly after 9 a.m. Father Christmas started his round of the wards distributing presents to each patient, while his retinue of clowns and animals, including a very amusing donkey and monkey, kept everyone, even the very ill patients, in roars of laughter with their antics. Kind friends had provided us with many good things, so that there was a useful gift for each patient.

Then came the dinners. Turkey and plum pudding! Turkey for everyone, who was able to eat it, thanks to the generosity of a member of committee who kindly offered to defray the extra expense, as it was a "Victory Christmas," a time to "eat and be merry"! Next came a little rest before the entertainments of the afternoon started. There were not the full complement of entertainers, but there were some troupes for every ward. "Punch and Judy," who has kept the children amused for many a year, was not able to come this year, as he was spending it in France. He did not forget us, though, as we heard that he was trying to get the leave due to him so that he would get to England in time to be at his usual post on Christmas Day. At 6 p.m. all the troupes and entertainers assembled in the receiving room, where they sang "God Save the King" and "Auld Lang Syne." Some of the troupes after this proceeded to the Out-Patient Department, where a dance was taking place for the maids. Here there was much merriment, as elsewhere. The maids were in fancy costume, and the gay scene was a striking contrast to the ordinary week-day assembly there. At 7.30 p.m. a real good Christmas dinner was provided by the Committee for the resident doctors and all the troupes who had helped to entertain the patients through the day. They sat down to dinner in the costumes they had worn during the day, and everybody was in excellent spirits, and they one and all agreed that the old-time Christmas was returning.

The Christmas festivities did not end here, however, as arrangements had been made to give a party in the Out-Patients' Department to 800 little sufferers who had attended there during the year. This was to take place on the Friday after Christmas Day. Through the kind help of the *Daily Mail*, we were able to again provide a good tea, an entertainment, and a present for each one. In each of the four corners of the big hall there was a large Christmas tree, and the hall itself was decorated with flags of all kinds. In the inner rooms tea was served to the little guests, cakes, buns, bread and butter, crackers, and other good things. Father Christmas and his procession of animals were there, too. The donkey and monkey (doctors dressed up) were a great source of amusement, to grown-ups as well as the little one. They did all sorts of antics, and when the monkey commenced to climb up a thin pipe up the wall the children did think they had a wonderful monkey. But that was not all, the monkey was next seen climbing across a girder in the roof. The children loved it, but the grown-ups held their breath until he safely reached the other side of the hall. The donkey meanwhile was enjoying himself amongst the guests, and no one knew what he would do next. He appeared here, there, and everywhere where he was least expected. And there were a lion, leopard, Polar bear, and, of course, animal trainers.

At 4.30 p.m. there was a tremendous outburst of applause, followed by the singing of "God Save the King," as her Majesty Queen Alexandra, H.M. Queen Maud of Norway, H.R.H. Princess Victoria, the Crown Prince of Norway, and the Hon. Charlotte Knollys arrived. In her ever gracious way Queen Alexandra spoke to all the wounded officers and shook hands with each one, after which the Royal party retired to the balcony in order to watch the entertainment. First there was a cinematograph—pictures of the German Fleet being handed over. How the children booed at some of the pictures and how they cheered when his Majesty the King and Admiral Beatty came on the screen! Then there was a picture of Charlie Chaplin, which all enjoyed, judging by the laughter one heard. Prince Olaf laughed as loudly as any. The Royal party remained for about an hour and a half, at the end of which they took their departure amidst a tremendous uproar of clapping and cheering. At 6 p.m. the "Animals" commenced to distribute the presents to the little ones, and as each child passed out they were given an orange. It had been arranged that their parents should call for them at this hour. The excitement was great, the little ones could not wait until they reached home to tell their mother, father, or big sister what a time they had had!

So ended a happy Christmas at "The London."

### The Middlesex Hospital.

"On earth armistice, good will towards 'white' men," is the keynote of this Christmas, whether in hospitals or elsewhere. At the Middlesex Hospital such a note of thankfulness and cheerfulness was struck, but in consonance with hospital conditions. During the day the resident staff laid aside their professional garb and decorum: a motley crew with a high-class barrel-organ went the rounds of the wards. Their prescription was mirth and merriment *ad lib*—a mental medicine as good as any tonic. In the afternoon a concert in the soldiers' largest ward was much appreciated. Neither were bodily needs neglected. Dinner in quality and quantity above the normal ration was enjoyed by all to whom it could be served with safety. Thanks to the thoughtful generosity of the honorary staff and many other friends of the hospital, the wards were brightly decorated; a Christmas tree with ample "fruit" on its branches gladdened the children's hearts; adults were cheered with fitting gifts; and the Overseas Red Cross Societies did not forget their wounded men. In short, everything was zealously done to relieve monotony, and care for the time was driven away. Sisters, nurses, staff, all worked with a will, and the gladness in the faces of those to whom they ministered afforded them a full reward for their untiring efforts. A word of tribute to the resident staff, whose musical and mimetic energies outside the walls earned a goodly sum from amused contributors for the funds of the hospital.

### The Pavilion General Hospital, Brighton.

Christmas began on Christmas Eve with an entertainment given by some of the sisters and V.A.D.s in the recreation room. A little play, "Between the Soup and the Savoury," was excellently acted. The whole entertainment was thoroughly enjoyed by a large audience of wounded soldiers. Just before the curtain rose a presentation was made to Colonel Coats, C.B., the distinguished veteran of the Afghan War, who, since the hospital came into existence, has devoted himself to organising the recreations of the wounded men in it and who is much beloved by all. It was not the first token of gratitude he has received from the men he has worked so hard for.

### OUR HOSPITALS' CHRISTMAS, 1918—(*continued*).

This Christmas gift, from the patients of "C" section, was a handsome tantalus.

On Christmas Day there was service in the chapel and thereafter an excellent Christmas dinner, the Government ration for which was supplemented by a welcome flock of turkeys, which, with other seasonable delicacies, was provided by generous subscription of the many good friends of the hospital that Brighton contains. During dinner Colonel Robinson, C.M.G., the A.D.M.S. the Sussex District, Colonel Maurice, C.M.G., the O.C. the Pavilion Hospital, the matron and others made a round of the wards and dining-halls. The decorations both in the Pavilion division and the York Place division were excellent and artistic and greatly admired. The O.C. took advantage of the opportunity to propose a vote of thanks to Mrs. Gordon Dill and the ladies associated with her, the ladies of the Red Cross, the ladies of the Green Cross, and the men of the Sussex Volunteers for the trouble they had taken to decorate and the trouble they were taking to help serve the dinners. The vote was heartily accorded.

In the evening Mr. Boardman and artistes of the Hippodrome, Brighton, gave a most charming entertainment to the men in the Pavilion. The men in the York Place division amused themselves with a whist-drive. The thanks of all are offered to the many good friends who worked and gave to make Christmas an enjoyable day in the hospital.

### War and Peace Scenes at Liverpool Royal Infirmary.

THE memory of "Peace Christmas" here will be treasured by all. Never has brightness and happiness been more abundant, accompanied by a genuine feeling of thankfulness and relief. The gifts in "kind," and the means to purchase them, were as usual too numerous to particularise; but it may be noted that Lord Derby sent Christmas-trees from Knowsley for each of the naval and military wards, as has been his custom during the war. On Christmas Eve a procession of nurses carrying coloured lanterns sang carols through each ward and corridor, the effect of which being both impressive and charming, and what a joy to the patients! Christmas Day commenced by a service and celebration in the chapel at 6 A.M., appreciated and attended by a large number of the nursing staff and patients. The chapel was as usual prettily decorated with holly and flowers. Dinners were served at noon, roast turkey, plum pudding, and mince pies appearing amid applause in each ward, the carving being done by members of the honorary and resident medical staff. Each patient had a useful gift, the soldiers and sailors receiving beautiful pigskin cigarette cases or a pouch with a pipe, accompanied by a serviceable garment.

The wards excelled in originality and tastefulness, each sister having given exceptional thought to her lamp shades. The sailors again showed a huge torpedo-boat destroyer, 5 feet 8 inches in length and perfect in every detail. In other parts of the ward could be seen an ingeniously made lighthouse, German submarines, and aircraft, while the lamp shades were "Peace Bells." The military wards made a special display of the Allied flags and bunting. Another ward had as its chief feature a miniature Egyptian desert, with its sandy plain, its oasis looking cool and fertile in one corner and a pyramid in the other, while dusty travellers on their camels are seen tracking their way across. On going to the next ward one begins to feel rather cold, for a snowstorm greets one in the doorway and quite a good "old-fashioned" Christmas scene is portrayed. Another ward had as its special attraction a park, with a cleverly arranged bandstand, a shelter, and a pond with swans, while at various corners were witty posters directing visitors to objects of interest. Flights of bluebirds were the particular note in one women's ward, while in another a pretty village green drew many admirers.

At 4.45 P.M. the resident and nursing staffs, the latter in quaint Old English costumes, provided short but excellent concerts in each ward, the audiences proving both appreciative and enthusiastic, thus bringing to an end one of the happiest Christmas Days within our memory, which was pervaded throughout by the feeling that matron, with her usual thoughtfulness, had been untiring in her efforts for the well-being of us all.

On Friday, from 3 to 5 P.M., each nurse was allowed to ask two friends as guests, tea being provided in the Home, followed by a tour of the wards afterwards. During the evening the nursing staff had a very enjoyable fancy dress party, prizes being given by ballot for the most original and prettiest costumes.

### Christmas in the Leicester Hospitals.

Make somebody happy to-day,
Some comfort and sympathy give,
And Christmas shall ne'er go away,
But always and ever shall live.

IT was in this spirit that the nursing staffs of the Leicester institutions entered upon their Christmas celebrations. A memorable time it has been, as it has been a memorable season throughout the world. For four long years the staffs of hospitals had been occupied in healing the ravages of warfare, in the relief of suffering and distress, working to the full extent of mind and body, when suddenly the constricting bands are removed in the knowledge that peace and goodwill have resumed their beneficent rule over mankind. Then the long pent-up desire to relax for the moment the discipline and the routine of duty makes itself felt, and Christmas opportunely provides the outlet through which expression can be given to the ecstatic feeling of being happy and making others happy. This feeling was not of course confined to nursing staffs, it was "in the air," and probably the patients, military and civil, possessed it in an even greater degree. That this was so was exemplified by the heartiness with which the patients entered into and, in many instances, devised the decorative effects. Never has the writer seen wards more daintily and effectively decorated, and never has greater enthusiasm been shown in the preparations. Wounded men have willingly given up their out-of-door time to lend a hand in cutting out ornamental patterns in paper, sisters and nurses have unhesitatingly sacrificed their off-duty time, and even made inroads on their after-duty leisure to supervise the decorative arrangements, and to rehearse for the carols and the "sideshows." The result has been a veritable time of enjoyment, which the arrival of a convoy of wounded on Christmas night only served to accentuate. The fare provided was generous and good, for even the autocratic Food Controller made himself as pleasant as we know him to be, and means were found of augmenting for the Christmas week those supplies which had been reduced almost to zero. The entertainments were on a similar generous scale, and the provision of comforts in the shape of an abundant supply of tobacco, cigars, cigarettes, and games was on an ample scale. The Mayor and Mayoress entered heartily into their visits to the various hospitals, where they were received with much cordiality. Probably the greatest interest was displayed in the military hospitals, and the knowledge that many of them would not

OUR HOSPITALS' CHRISTMAS, 1918—(*continued*).

be open as such for another festive Christmas only made the desire keener to make the occasion a "red-letter" period. The officer commanding the 5th Northern General Hospital (Brigadier-Colonel L. K. Harrison) must have been the busiest and the most happy of Father Christmases, and his arrangements were most effective, and no happier Christmas has ever been spent at the hospitals and institutions under his supervision. At the Royal Infirmary the same happiness prevailed, and the arrangements made by Miss Vincent, the matron, met with the utmost approval on the part of patients and visitors alike. The assistant matron (Miss Marriott) and the sisters and nursing staff deserve a generous tribute of praise for their enthusiasm and enterprise. They took upon themselves the entire onus of the entertainments, and it must be a source of great joy to them that, without a single outside helper, they carried through an entire programme which was repeated in every ward. The "Toy Symphony" was greatly appreciated. The representations of land workers were very effective, and the improvised "cattle" which they pushed or led as fancy dictated, added greatly to the general merriment. A fine bullock was "fed by the High Sheriff of Leicester," a compliment to the institution's chairman, Mr. T. Fielding Johnson, Jun., who was present at more than one of the performances. Perhaps, however, it was in the series of Elizabethan dances in costume that the troupe excelled, and they met with a great reception as they afterwards in a figure dance proceeded the entire round of each ward to light and gay music. Another pleasing instance of the spirit of the nursing staff was exhibited on Christmas night, when about sixty of the staff paid visits to each ward for carol singing, the lights in the wards being extinguished, and each nurse being provided with a lighted candle to make the procession more effective.

At the Gilroes Hospital, the Workhouse, and at other civil institutions, the spirit of Christmas was well sustained, and patients everywhere were delighted with the arrangements made for their enjoyment.

No reference to the Christmas festivities would, however, be complete without special mention of the Leicester War Hospitals Committee, which has been aptly described as the Fairy Godfather of the wounded soldiers, by Col. Harrison. Thanks to the initiative of Councillor Squire and Mr. W. G. Gibbs, the hon. secretary, a bountiful supply of tobacco, cigarettes, games, and other comforts was provided. This committee has throughout the period of the war supplied comforts for approximately 70,000 soldiers who have passed through the military hospitals as patients, and whose recovery in the words of Col. Harrison, has been materially assisted by the unlimited provision. The importance and extent of the work can be judged by the fact that for the fourteen Leicester war hospitals alone 9,000lb. of tobacco, and nine million cigarettes (secured free of duty through the initiative of the society in the first year of the war), 70,000 boxes of matches, writing-pads, paper and stationery, billiard-tables, pianos, books, magazines, newspapers, games, gramophones and gramophone fittings and supplies, playing-cards, and numerous other gifts have been supplied and distributed in large quantities without charge to the various institutions. When to these supplies are added those to the fifty-two hospitals and institutions associated with the 5th Northern General Hospital and for the Leicestershire Prisoners of War Fund, a full idea of the scope of the committee's work and responsibility is provided. In the last year alone ten tons of cigarettes, three tons of tobacco, and 3,600 cigars were cleared from the Duty Free Customs Depôt. The committee has further rendered indispensable service in the clearance of six tons of tea, ten tons of sugar 3cwt. of chocolate, and 13¼ cwt. of cocoa, duty free. The total expenditure amounted to approximately £3,000 in the last year, an amount which has been raised by an Alexandra Day collection, and by contributions from friends and supporters, workpeople's collections, etc. In all this work during the four years of war the ex-Mayor and Mayoress (Alderman Jonathan North and Mrs. North) were greatly interested, and their influence and support in all service for the war hospitals deserves acknowledgment.

### Salisbury Infirmary.

DESPITE an undue amount of illness in the wards and amongst the staff owing to influenza Christmas was very happily spent by military and civilian patients alike. Some soldiers went on holiday leave, but many remained to assist in merry mood with the decorations and to enjoy the provision made for those who were not considered fit to travel. Beautiful evergreens and flowers, and many flags decked the wards, and much variety was shown in the decorations. Early on Christmas morning nurses sang Christmas hymns before proceeding to early service. Many attended the Celebrations of Holy Communion in the chapel, and services were held in the wards for patients. At breakfast-time each member of the nursing or domestic staff found a useful gift on her plate. Later in the morning Father Christmas, personated by one of the house surgeons and attended by six girl Guides, made a tour of the wards, and presented each patient with a parcel containing useful gifts. Next in order came the great event of the day—the Christmas dinner. Roast turkeys and fowls were carved by members of the visiting staff or the residents. Christmas puddings pronounced to be as good as pre-war ones, were much enjoyed by all. In the afternoon each patient received two visitors, for whom tea was provided, making large tea parties in each ward. Many visitors interested in the institution made a tour of the wards including the Bishop of Salisbury, General and Lady Slater, the Mayor and Mayoress, and the chairman. A concert party arranged by the house surgeons gave selections of music during the afternoon, and in the evening nurses and maids sang carols, conducted by the Chaplain. On Friday, the 27th, the nurses made merry over a fancy-dress supper and dance, and on Tuesday, the 31st, an entertainment was given by the night nurses in the Recreation Room to the patients, and a festive supper was provided. A very generous response was given to the Christmas Appeal sent out by the matron and Chaplain, so that all extra expenses have been well covered.

(To be continued next week.)

